# Employing Natural Language Processing as Artificial Intelligence for Analyzing Consumer Opinion Toward Advertisement

**DOI:** 10.3389/fpsyg.2022.856663

**Published:** 2022-05-31

**Authors:** Huilin Sun, Muhammad Zeeshan Zafar, Naveed Hasan

**Affiliations:** ^1^Business School, Shandong Jianzhu University, Jinan, China; ^2^School of Business Management, University Utara Malaysia, Sintok, Malaysia; ^3^Faculty of Management and Administrative Sciences, University of Gujrat, Gujrat, Pakistan

**Keywords:** attention, emotion, cognition, qualitative research, tweets, Pakistan, Think-aloud, product advertisement

## Abstract

With the advent and integration of technology in business, marketers started investing in numerous media platforms to influence the consumer's sentiments. Artificial intelligence has been proved as one of the innovative tools of digitalization to change consumer's media habits. Owing to the growing trends of e-commerce, the traditional advertising model is insufficient. Therefore, advertisers are taking advantage of artificial intelligence technology to meet current requirements. Thus, a deeper understanding of product advertisement with reference to consumer sentiments and its implications need to be established. The current research depicts the contribution of artificial intelligence to analyze the consumers' attention, cognition, and emotion. The target product was Samsung Galaxy. Researcher of the current study has employed Think-aloud procedure for data analysis. Tweets dataset was divided into 2 categories. For international consumers' sentiments 30,877 tweets whereas for Pakistani consumers' sentiments tweets dataset was 26,834. For data analysis, authors used Nvivo for generating theme. The Nvivo produced word cloud. The word cloud generated with Pakistani tweets revealed that consumer attachment with Samsung product is based on emotional and attention and the preferred features of Samsung products are linked with emotional and attention. In contrary to that rest of the world tweets unfolded that emotion, attention, and cognition make consumer preferences while selecting Samsung products. This study is useful to the cellular companies for targeting across the world population. The consumer preference varies while selecting cell phones. This study will provide a better idea to cell phone companies for manufacturing consumer oriented cell phones to get better results. Moreover, future research should add more countries separate data and generate a comparative study between developed countries consumer and developing countries consumer preferences. In addition to companies with better insights of consumer can highlight the most attractive features of cell phone in their advertisements.

## Introduction

Human being is a “Homo Sapiens” which means a “wise-man” having some specific capabilities such as thinking, reasoning, acting, and learning and to behave (Lee and Cho, 2020). Besides, these human capabilities make them able to understand their environment and act accordingly. Reciprocally, this ability of humans is explained by a word “intelligence” which is borrowed from Latin word which means “To Understand” (Kumar and Sha, 2017). It was 1956, when the word “Artificial Intelligence” (AI) was first time utter in a conference (Russell and Norvig, 1995; Martínez-López and Casillas, 2013). At the initial stage the question was to bring human brain ability into a machine system which could behave like human mind. Although some of the technologies like home appliances and motors have claimed to be intelligent but these devices do not have capability of reasoning, perception, action and behavior (Freier, 2016). Additionally, the concept of AI was the beginning of robotics (Perez et al., 2018). Later on, several fields, such as business, medical science, agriculture, and construction, have rendered the services of AI to serve their target audience (Ribeiro et al., 2021).

It is not possible for business world to ignore the decisiveness of AI to target their customers. There are multiple tasks in business, which need valuable and automated solution for their success (Negnevitsky and Intelligence, 2005). To forecast future, business executives need *ad-hoc* intelligence system (Martínez-López and Casillas, 2009; Casillas et al., 2012; Chen et al., 2021). In addition to the potentialities, AI assist companies in strategic intelligence such as managing customer relationship, positioning products, segmenting the market, designing personalized advertisements, and product-harm crisis (Martínez-López and Casillas, 2013; Dubey et al., 2021). To understand consumer is very complex because there are numerous channels and forms through which they express their needs and attitudes (Kietzmann et al., 2018). Companies demand technological innovations to conduct advertising tasks (Qin and Jiang, 2019). The transformation of business model from physical to online has also changed the advertising method. Therefore, for effective communication and marketing, companies are paying much attention on online brand communality engagement OCBE (de Almeida et al., 2018). Consumer's unstructured and freely written online comments at various blogs like Twitters, Facebook and other social media channels are very valuable for companies in improving their products. The application of AI in advertising has emerged the concept of “smart advertising” (Vakratsas and Wang, 2020). AI technology has restructured the advertising process (Qin and Jiang, 2019). It is not confined to design advertisement but to understand the consumers' emotions and cognitions toward advertisements contents (Abbas et al., 2018).

The advertising cost is increasing worldwide. It has been observed that on global digital advertisement, the estimated cost has risen to 17.6% (Lee and Cho, 2020). Therefore, to cut short the ineffective advertisement there is a need to understand consumer attention, cognition, and emotion toward advertisements. Companies are trying to figure out solutions for effective advertisements by using big data like social media comments. Aforementioned research has suggested investigating the consumer insight for designing consumer oriented advertisement (Qin and Jiang, 2019). Therefore, the consumer life-style and behavior is imperatively essential to be understood (Li et al., 2021). Whereas, gaining the understanding about the consumer life-style and behavior is almost impossible without the use of innovative technological tool like AI (Di Vaio et al., 2020). Owing to the increasing cost of advertisement and rapid failure of advertising contents have increased companies' unnecessary financial budget. Moreover, to get competitive edge the consumer insights is inevitable. As Shrivastava (2014) stated that 1 of the extensively accepted source of information for the consumers is advertisement, that's why choice of media is pivotal for the marketers.

Additionally, consumers' online feedback exhibits their true representation as it has based on their experiences. Eventually, the objective of this study is to understand consumer attention, cognition and emotion toward advertisements by using big data sources. Extracting the online consumer content helps the decision makers to analyze the customers' sentiments and to haul out the required information. AI can help in data gathering and affective computing for sentimental analysis, which ultimately helps marketers to design the effective customer satisfaction strategies but also to manage their employee engagement (Khrais, 2020).

## Literature

A profound revolution has been observed in advertising. Marketers must face and embrace the new changes while conducting advertisement campaign to reach and communicate product features to customers. The composition of advertisement in digital era has transformed and advertisers are shifted from electronic and print media to social media. Advertising researchers and practitioners have raised a question regarding changing nature of advertisement “will we continue to follow the same pathways and trace the same footprints of those who have gone before us, or will we set off on a new path, embracing change and acknowledging that new approaches are needed?” (Schultz and Schultz, 2016). Advertiser generates various appeals while targeting customers such as cognitive, emotion, and attention. The nature of product defines which appeal is suitable for delivering product information. The extraction of consumers' emotion, cognition, and attention from their online comments is not an easy task. These cognitions and emotions are drivers to information and message processing that leads to formation of attitude (Zaki et al., 2021). The availability of big data in the form of consumer feedback pertaining to the companies' advertisement must utilize for consumer insight. To manage unstructured online data, there is need of systematically programed software which can read human language and transformed it into usable form.

### Advertisement and Consumer Attention

The objective of effective advertisement is to grab consumer attention because it demonstrates the consumer attitude and behavior toward products (Haase et al., 2020). Aforementioned study has reported that for an advertisement, the primary challenge is to raise the consumer attention (Haan and Moraga-González, 2011; Dankwa, 2021). Advertising concept is in a transitional phase, from offline to online, to grasp consumer attention (Chandrasekaran et al., 2018). Moreover, the growing concept of advertisement is consumer voluntarily seek out relevant information (Rayport, 2013). Therefore, advertisers should understand the mechanism for designing creative and attractive advertisement for consumer attention (Rosengren and Dahlén, 2013). In present competitive environment, it is an easy task to gain consumer attention because most often consumer gets confused in extracting relevant information (Jiang et al., 2017). Past literature has reported that the percentage of ignored advertisement from consumer is 30% (Lipsman et al., 2013). The cause of this noticeable percentage is lack of proper allocation of information whichis easily visible to target consumers (Vranica, 2013). The technological innovations have changed the consumer access for searching product related information. The digital advertisement has made the product related information available at consumer door step. The dynamics of advertisements has changed with the introduction of Facebook ads, YouTube ads, and various blogs. Consumer can instantly extract information and response. Furthermore, advertisers should focus on personalized advertisement for the attention of consumers rather generic contents. The technological advancements have removed the boundaries and social media has made it possible to reach the customers around the globe (Deng et al., 2019). The online behavioral advertising has unfolded the new method to access consumers (Chen and Stallaert, 2014). The huge availability of unstructured information, for instance Facebook comments, You Tube comments etc. is a big hurdle for advertiser to design consumer centric advertisement for best consumer attention. Past studies have reported that nature of advertising is changing and the involvement of individual communication is rapidly increasing (Kumar and Gupta, 2016). Companies are designing ads according to the behavior and needs of consumers (Schultz and Schultz, 2016). Moreover, for consumer attention the future of advertising is personalized targeting (Rust, 2016).

*P1: Advertisement develop consumer attention regarding any product*.

### Advertisement and Consumer Cognition

To understand advertising effect on consumer is a challenging task for advertisers (Bagwell, 2007). The role of advertisement is two-fold, 1 is to provide information to consumer pertaining to the seller and product price and quality, second identify consumer needs and taste (Honka et al., 2017). Moreover, the available information in advisement shapes consumer cognitive abilities toward selection of product. Cognition is the process of acquiring information and understanding with the help of senses, thoughts and experiences which ultimately results in consumer behavior, judgment, and decision making (Wörfel, 2021). Advertisement provides exposure to consumer and enlightens consumer thoughts for informed choice (Adamopoulos et al., 2018). Advertisement is a source of awareness (Zhou et al., 2016). Furthermore, for a single product an individual is interacting with multiple advertisements. These multiple advertisements informing consumers as well as increase the information process of consumers for better options (Ali, 2021). Moreover, consumer is unable to take rational decision without the assistance of marketers and advertisers (Bartels and Johnson, 2015). In addition to due to technological enhancement advertisers have multiple sources to target consumer mind. The emerging source is online advertisement or digital advertisement (Hof, 2014). These digital advertisements have shifted advertisers from massive advertisement to personalized contents in advertisement (Bang and Wojdynski, 2016). The consumer centric advertisement is effective for better response. It reduces consumer information processing efforts for the selection of any product (Bleier and Eisenbeiss, 2015). Owing to the increasing budget of advertisement the advertisers are trying to figure out effective and efficient sources to reach consumers' mind. Furthermore, for the success of any advertisement the understanding of customer thought is decisive (Pareek et al., 2022). The new platform of advertisement like online ads has provided instant interaction with consumers. Online ads are actually an interface which facilitate consumer for ads feedback and to companies for consumer insight. The modern methods of advertisement have increased consumer involvement in search of product information. The increasing trend of customers' disclosure has also accelerated customer feedback trends. The unstructured customer data can provide fruitful results for customer insights as well as designing customer centric advertisements.

*P2: Advertisement affects consumer cognition while selecting any product*.

### Advertisement and Consumer Emotion

Advertising effects are many folds such as physiological effects, creating positive effect, and emotional effect. The advertisement creates reaction among consumer (Chang, 2019). These reactions based on the objective of advertisement. Companies conduct advertisement campaign as per the nature of products as well as which feature they want to promote to target the need of consumer. Study has reported that according to advertising practitioner's emotions are the most the most decisive element (Bakalash and Riemer, 2013). Word of mouth advertisement also effects consumers' emotions and choices (Miremadi et al., 2021). There are two schools of thoughts pertaining to the emotions, one the integral emotion and second incidental emotion (Achar et al., 2016). Integral emotion evoked by messages in advertisement which are strategically or deliberately embedded by advertiser and incidental emotion provoked by circumstances. Moreover, the advertiser most often integrates both integral emotion and incidental emotion while designing an advertisement (Choi et al., 2016). The dramatic changes in media environment have opened various platforms for advertisers to reach customers and to evaluate customer emotions and feels toward the selection of a product (Malthouse and Li, 2017; Murtarelli et al., 2021). Moreover, the advertisement arouses various emotions among viewers such as fear, sad, and happiness which reflect different state of mind of an individual (Das et al., 2015). It has also been reported in aforementioned studies that the behavioral process of an individual can be influenced by emotional reaction without cognitive involvement such as thinking, reasoning. and conscientiousness (LeDoux, 1996; Vermeulen and Beukeboom, 2016).

*P3: Advertisement affects consumer emotion while selecting any product*.

### Artificial Intelligence for Consumer Attention, Cognition, and Emotion in Advertising

Haenlein and Kaplan (2019) define AI as “a system's ability to correctly interpret external data, to learn from such data, and to use those learning to achieve specific goals and tasks through flexible adaptation”. The involvement of artificial intelligence and various big data analytical techniques have removed the media boundaries to reach target customers (Malthouse and Li, 2017). The artificial intelligence technology has also transformed the advertising methods (Kietzmann et al., 2018). Advertisers are shifting advertising contents from advertising-centric to personalized contents (Kumar and Gupta, 2016). The cause of this shift is consumers' insight pertaining to their attention, cognition and emotion toward advertising (Liu et al., 2017). The consumer starts his journey with need identification and the need recognition mobilize consumer through various stages such as consideration, evaluation, purchase, and post purchase (Court et al., 2009). In addition to the introduction of internet has facilitated consumers to interact with advertisement and give instant feedback. The nature of online advertising is interactive which accommodate consumers to conveniently give and seek information pertaining to product experience (Prendergast et al., 2010). Aforementioned studies have observed the skyrocketing rise of social media trends which is providing unlimited access of information to consumer regarding products and brand choices (Chu and Sung, 2015; Araujo et al., 2017; Yoon et al., 2017). The online information effects consumer purchase intention and decision. These online feedbacks have generated big data which can be utilized for designing consumer-centric advertisement. Moreover, marketers are operating in digitalized environment which is an extended avenue to reach customers (Kumar and Gupta, 2016; Schultz and Schultz, 2016; Becker et al., 2017). The consumer written expression in the form of online feedback comprised of individual's cognitive, emotional and attention toward a product. Although the excessive available online data about consumers' opinion toward product is very effective and helpful for the insight of consumer but the utilization of data to achieve organizational goal needs an intelligence system. The artificial intelligence has introduced the concept of programmatic advertising (Chen et al., 2019). The programmatic advertising assists companies in understanding consumer online behavior. Moreover, programmatic advertising is an emerging phenomenon (Chen et al., 2019). According to Busch (2016) programmatic advertising is “the automated serving of digital advertisements in real time based on individual advertisement impression opportunities”. In addition to in programmatic advertising the advertiser targets right people at the right time instead of “spray and pray” advertising.

*P3: Artificial Intelligence can effectively evaluate consumer attention, emotion and cognition regarding any product*.

## Theoretical Framework

The current model has underpinned with cognitive theory. According to Jean Piaget's theory of cognitive, the cognitive theory is characterized by “their focus on the idea that how and what people think leads to the arousal of emotions and that certain thoughts and beliefs lead to disturbed emotions and behaviors and others lead to healthy emotions and adaptive behavior”. The individual share their thoughts and feeling with complete freedom of expression at their tweeters accounts. These comments regarding any object facilitate scholars to evaluate individual's positive and negative opinions. Therefore, the objective of the current study is to evaluate individual's emotions, cognition and attention for purchasing a mobile product, which is facing defamation issue. The selection of such products has required a strong emotional, cognitive, and attention.

## Methodology

In consumer behavior the qualitative research method has become essential because of its classical advantages in dealing with big data and data mining (Petrescu and Lauer, 2017). Moreover, the numerous sources of big data have the data collection processes. For consumers' insight, researchers and practitioners are more interested into the techniques, which can handle big data rather using traditional close-ended questionnaires. Social media and companies' official blogs are not only the source of big data but also generate neutral opinions with freedom of expressions.

This research has utilized the Shapiro's (1994) Think-aloud procedure to study the consumer insights. In this procedure, individuals have two ways of expressing their opinions; dialogue and writing. Consumers usually use different mediums to express their sentiments such as Facebook, twitter, etc. For this research, twitter's data set was taken into account for analysis. Aforementioned studies witnessed that twitters and Facebook are convenient, easy, and cheap social media methods for generating massive word of mouth for both positive and negative (Javornik et al., 2020). Therefore, authors of the study involved twits data for analyzing consumer opinions. Besides, authors have evaluated consumer's twits for Samsung Galaxy. The purpose to select Samsung Galaxy was an incident related to Samsung Galaxy Note 7 when in 2016 news spread in the market that Samsung Galaxy Note 7's battery exploded and US flights banned the product (Krisher, 2019). This was the product-harm crisis situation. Later, Samsung has fixed the problem and re-launch the product and also introduced Galaxy Note 8. Nevertheless there was a fear which has prevailed in the market for a time being. Now authors of the study interested to understand consumers' opinions about Samsung Galaxy with respect to product features, brand loyalty, and product image by using individual's sentiment's proxy like emotion, cognition, and attention. There are some official websites which maintained twitters' dataset formally and used for research analysis like GitHub and Data, World, etc. Authors have divided the twits into two sections like international tweets which comprised 30877 and Pakistani consumers' tweets dataset which was 26834. Tweets were analyzed using natural language processing software Nvivo 12 and generated word clouds. In this research authors' focused on the data available from 2016 to 2018. The data was further classified into two categories (i) 2016 to 2017 and (ii) 2017 to 2018 to have a better understanding about the consumer's changing sentiments.

## Analysis

### International Tweets

The written opinions were based on consumers' sentiments about Samsung Galaxy. Besides, these consumers' comments also represent their attention, cognition, and emotion about the product. From 2011 to 2014 total tweets were 14877 in dataset. The data was analyzed using natural language processing with the help of Nvivo 12 software. Additionally, the procedure adopted for generating word could was based on display of word and minimum length of word exist in tweets. The author used 100 for display of words, which are most commonly used in tweets. Secondly, to avoid any unnecessary display of word in word cloud authors fix the limit of 5 which mean that display the only words which consists of 5 letters. The variables used in research were having more than 5 letters like attention, emotion and cognition. Moreover, Nvivo 12 software provide grouping options to researchers like exact matches, with stemmed word, with synonyms, with specialization and with generalization. Therefore, the authors of the study opted with synonyms to explore the words, which are linked with emotion, cognition, and attention.

**Figure 1:**
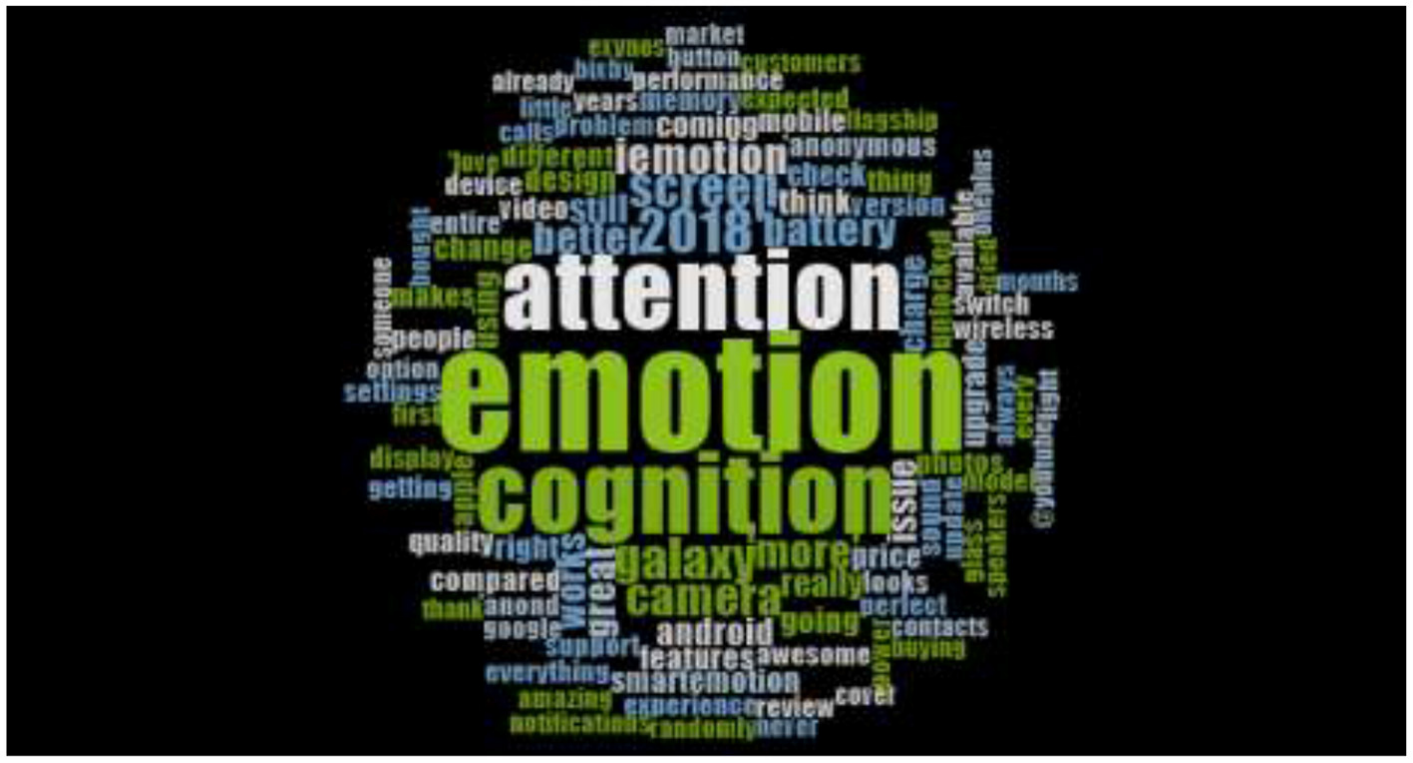
Across the globe Consumer's Tweets word cloud for Samsung Galaxy (2016–2017).

Nvivo 12 has generated word cloud, which is the graphical representation of words. The word cloud of current study generated with the words was having similar meaning. Word cloud has been recognized with another name tag cloud is actually visual representation of the unstructured text data. The words interpreted with their size and color. Moreover, unstructured data which is in the form of text continue to see unprecedented growth within the field of social media. Therefore, there is a need to analyze the data, which is available in massive amount. Figure 1 is describing the word cloud of data representing to the 14877 tweets of consumers regarding post purchase opinions for Samsung Galaxy. Besides, the most highlighted words like attention, cognition, and emotions are the words which are frequently used in tweets. It reveals that consumers' opinions in their tweets are positively link with Samsung Galaxy. The tweets dataset was converted into 3 nodes attentions, emotion and cognitions. Furthermore, based on the origin of the codes authors developed themes depending upon the similarities and difference in the meaning of notions under emotion, cognition, and attention to achieve the objective of the study.

**Figure 2:**
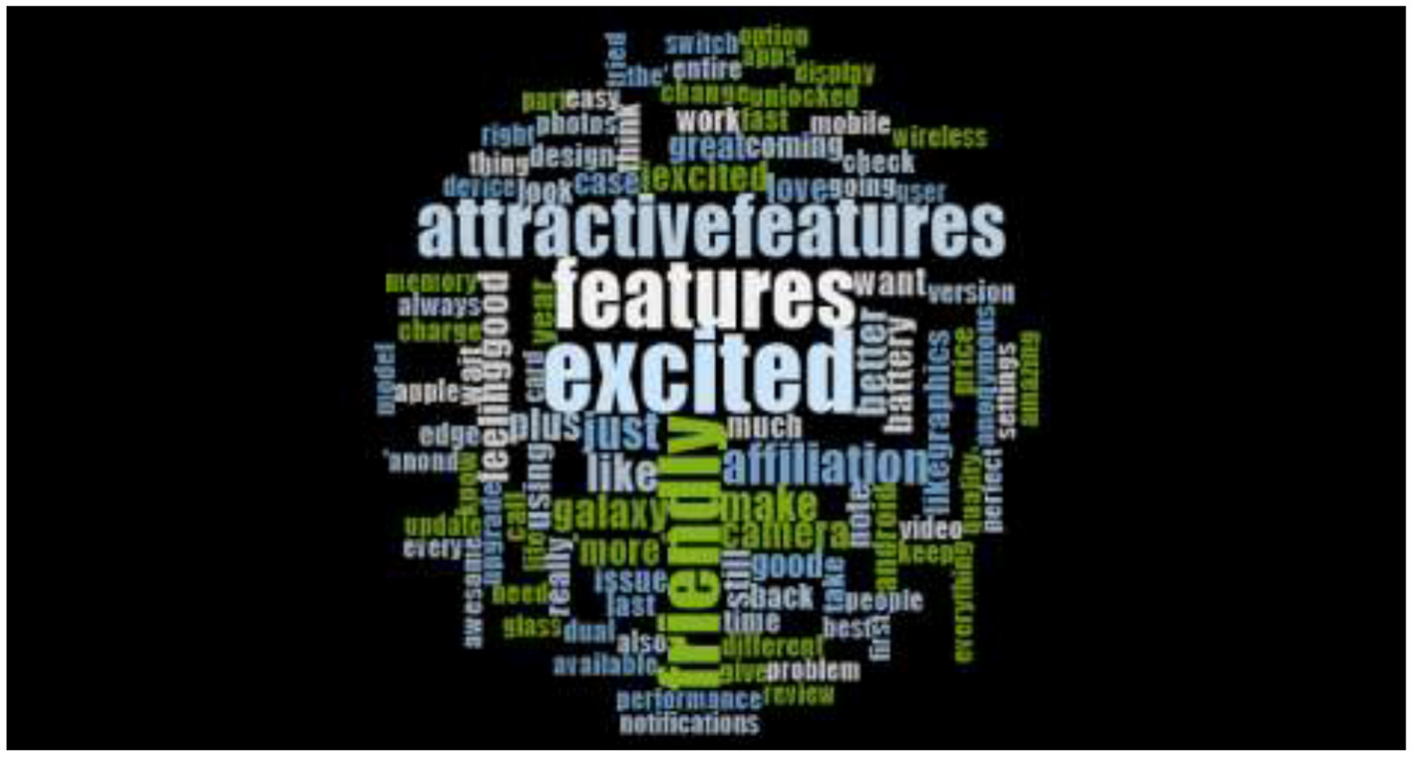
Across the globe Consumer's Tweets word cloud for Samsung Galaxy (2017–2018).

The prominent words of the word cloud figure are the attractive features, excited and friendly. These words are describing individual's sentiments like emotion, cognition, and attention. Moreover, the rest of the small word, which are scattered around the prominent words also indicating consumer's sentiments like quality, features, price, and change. Figure 2 is representing the word cloud of tweets dataset from 2017 to 2018 and total tweets were 16000. The word cloud prominent words have indicated that consumers are more interested in Samsung Galaxy's features and are more excited in expressing their thoughts and emotional affiliation with the product.

**Figure 3:**
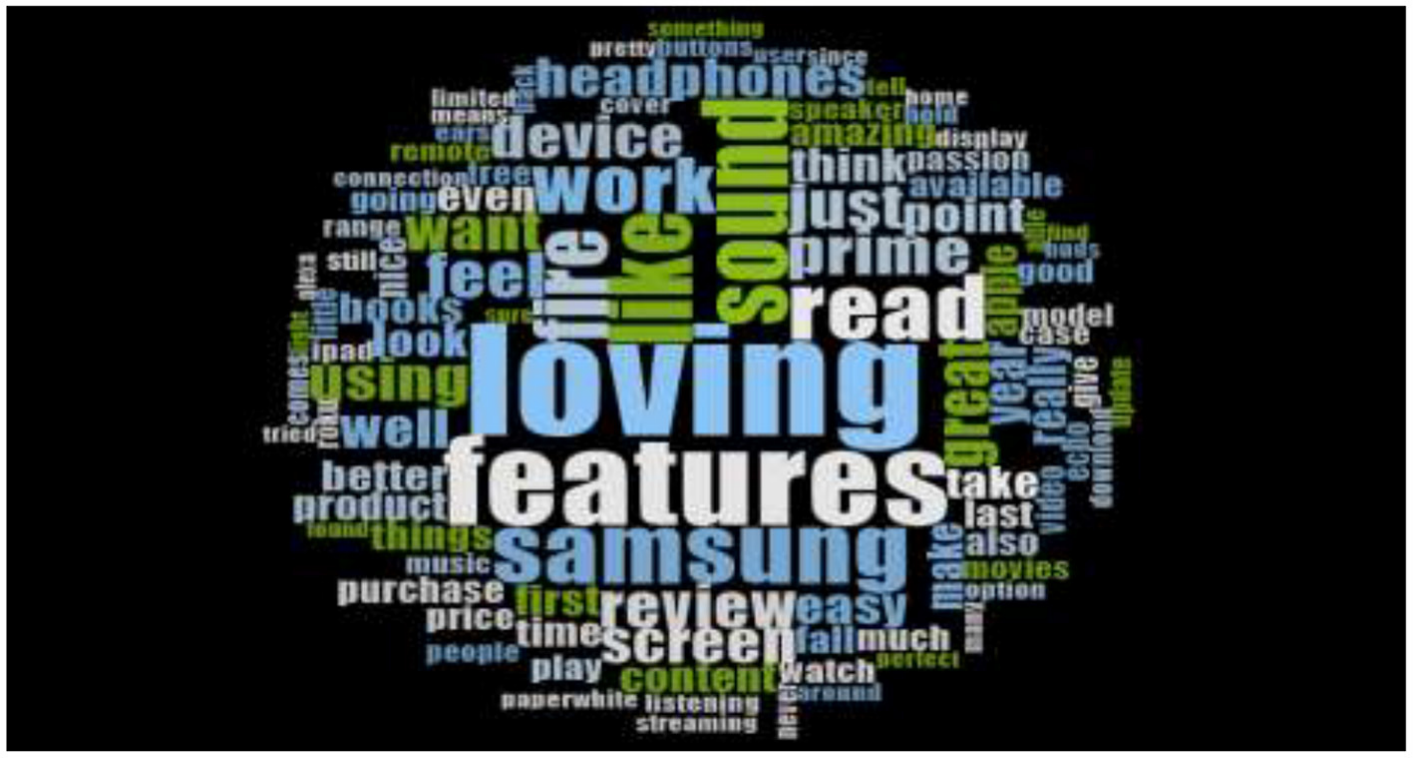
Pakistani consumer's sentiments about Samsung galaxy (Tweets Years 2016–2017).

**Figure 4:**
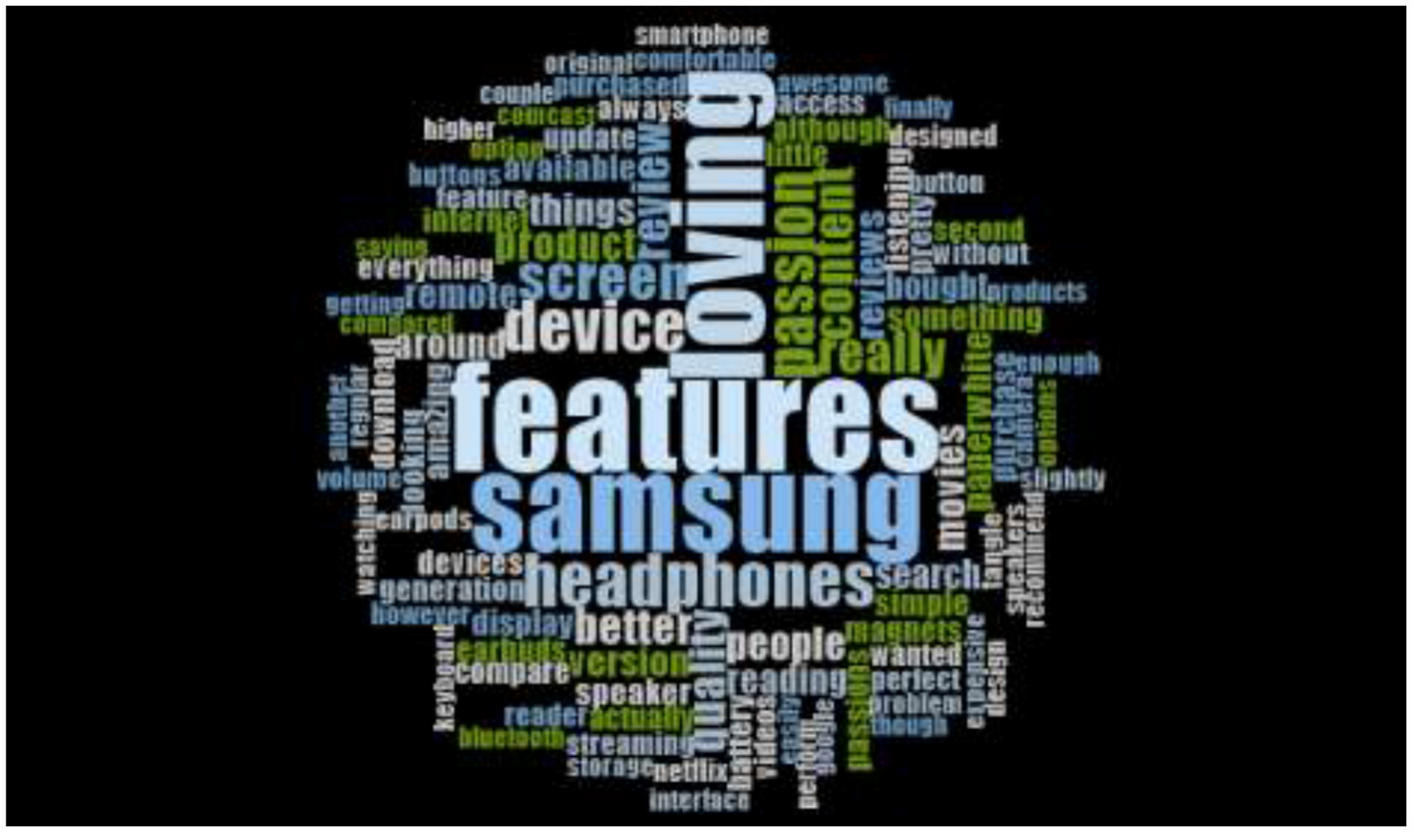
Pakistani consumer's sentiments about Samsung galaxy (Tweets Years 2017–2018).

Moreover, Figures 3, 4 are the word cloud, which has generated by using the tweets posted by consumers' at their twitter's account regarding their opinions toward Samsung Galaxy. The word cloud reveals that consumer posting positive remarks and feedback regarding Samsung galaxy because the most prominent words are loving and features. Besides, the surrounded words like easy, better product, passion and amazing are also demonstrating the positive cognition, emotion, and attraction of consumers toward Samsung Galaxy.

### All Tweets

**Figure 5:**
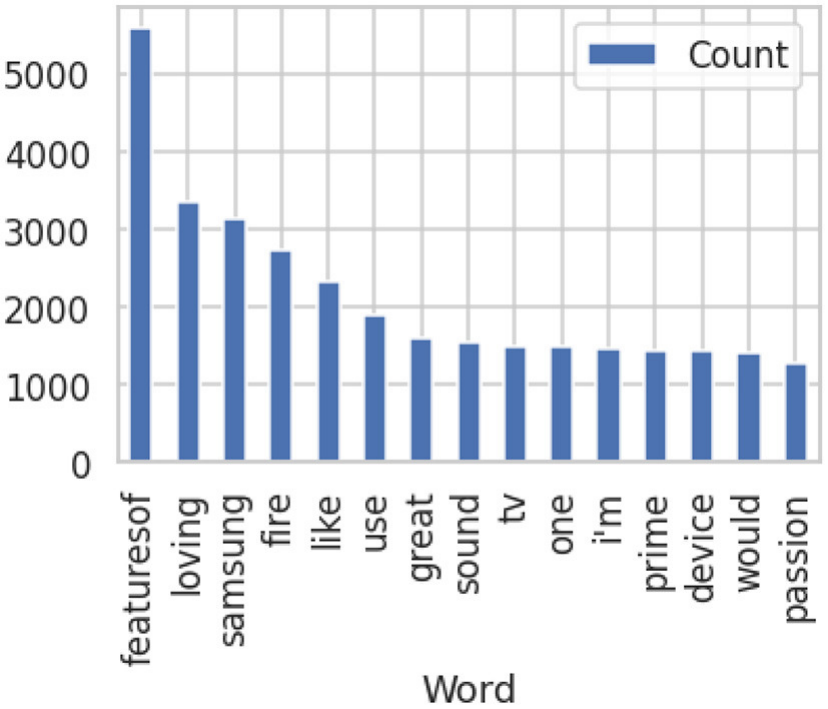
Prominent words in data set after pre-processing tweets with NLP.

The majority of the words are representing the consumers' emotions and cognition such as loving, passionate, awesome, features, comfortable, amazing, etc. The prominent words originated from the data are love, amazing, features, headphones, and contents, great and better product as can be seen in Figure 5 from the tweets posted from 2016 to 2018.

From the above graph we can conclude that for Pakistani customers find the features of mobile phone are more attractive. Therefore, in Pakistan the satisfaction level of customers can be boosted by emphasizing on mobile features in advertisement. The dataset also revealed that customer behavior and life are also playing an important role in developing customer's sentiments for the product. Using Artificial intelligence for sentiment analysis we can also identify these reasons at the back of customer opinion building in Pakistan to get a deeper understanding related to research focus.

**Figure 6:**
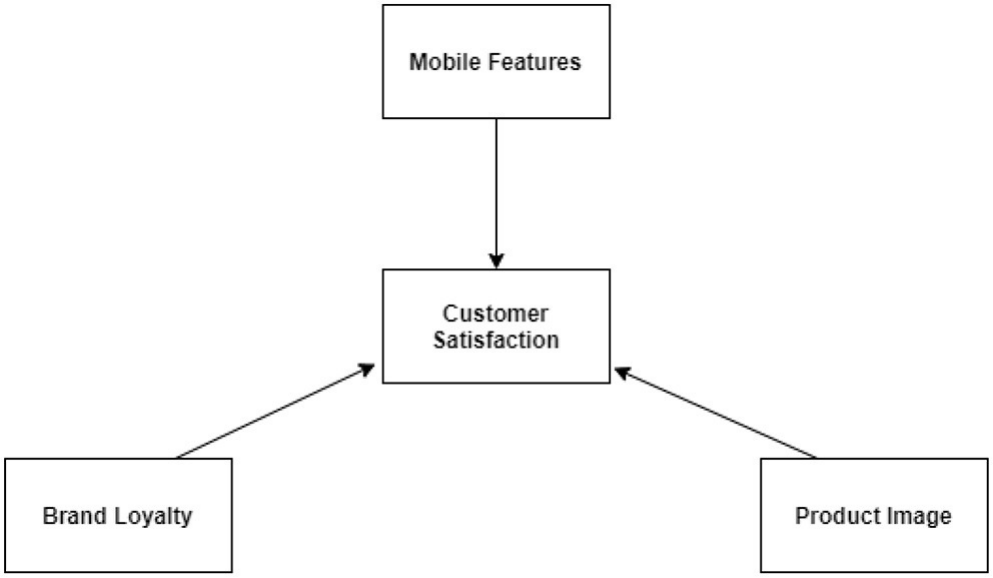
Factor affecting customer satisfaction.

These findings and factors influencing customer satisfaction as can be seen in Figure 6 are in alignment with the present study's findings where personal bias, brand image, and specification is known to play a key role in purchase and interest. Extracting factors such as these from customer comments can also better guide advertisers to design appealing advertisement for their target customers Most often consumer expresses their actual purchase behavior and post purchase behavior about product after getting influenced by such factors. These positive and negative opinions with influential factors as contributors, which are creating and motivating these opinions when identified through Natural language processing can speed up the advertising processes with respect to consumer's attention, cognition, and emotions.

## Discussion

The objective of the study was to analyze consumers' unstructured opinions to get their insights toward an advertisement. Author of the study has employed twitters' dataset regarding Samsung Galaxy. Moreover, for the data analysis, natural language process (NLP) was used named Nvivo 12. The tweets were comprised of consumers' 3 different sentiments like emotion, cognition and attention. Owing to the increasing competition companies have very limited options to get competitive edge even in highly innovative products like cell phones. Furthermore, for value addition companies should understand consumer's preferences. Companies stimulate consumer's need with advertisements and these advertisements are the source to target consumer's emotion, grab consumer's attention and strike consumer's cognitive behavior. To collect consumer's feedback with structured and self-administered questionnaire make consumer confined in limited options. Therefore, the involvement of big data and algorithm to evaluate consumers' extensive opinions make significant difference as compared to traditional method of data collection. The involvement of big data and algorithm are the techniques of artificial intelligence to transform the unstructured and meaningless data into meaningful themes. Artificial intelligence has shifted the method to advertise and guide consumers. Moreover, for consumer-generated data mining various techniques of artificial intelligence are play vital role. Researchers and practitioners are suggesting using big data for designing consumer oriented advertisements. There is a need of ad- hock intelligence system, which can assist individual to evaluate and interpret consumer's post-purchase unstructured expression.

The interpretation of consumers' responses on Facebook and twitters can cause the success of advertisement. These social media platforms are the best source for consumer to express their post purchase opinions. The results of current study have unfolded the fact that consumer's share their emotions, attention and cognitive behavior in their tweets which are prominently appear in word cloud. These online opinions are also an electronic word of mouth (e-WOM) and play as an advertisement for potential customers (Araujo et al., 2017; Miremadi et al., 2021). Aforementioned studies have suggested that to succeed in market and to design effective advertisement the advertiser should frequently monitor consumers' electronic unstructured opinions (Colliander et al., 2015; Craig et al., 2015). The unstructured consumer's data is the window of opportunity for advertisers, researchers, practitioners, and policy makers to target consumers with customized advertisement (Chu and Kim, 2018). In addition to machine leaning, assisting advertisers to collect consumer data from multiple sources and mine them to deliver on spot consumer insights.

## Future Direction

The current study has some limitations and author has suggestions for future research. The tweets dataset of the current study was taken from GitHub.com and dataset. In future it is better to conduct an in depth interviews by involving consumers who are using various cell phones. The data collected from in depth interviews should be compare with online posted tweets. Tweets also have some limitations like word count and most often consumer are unable to express their sentiments in detail. Correlation can also be used to measure and describe the relationship between opinions and factors affecting these opinions. Because, it can also be used for prediction as when 2 variables are correlated, then 1 variable can be used to predict the other variable through artificial intelligence and machine learning. This can give us a more accurate analysis of the twitter data and its values. Moreover, in tweets dataset it is not possible to differentiate that which users are sharing their own experience and which are just endorsing others opinions. Furthermore, although NLP Nvivo 12 generates themes from unstructured and meaningless sentences nevertheless in future researchers should involve more sophisticated AI software for data analysis.

## Conclusion

The concept of artificial intelligence has inculcated in computer science and medical science. Additionally, the artificial intelligence is also serving to business while taking various recruitment decisions, marketing decisions and for reviewing advertisement feedbacks. Owing to the presence of digital environment customers are very much accustomed with and experiencing digital advertisement. Moreover, the current study is a small contribution in existing literature which is opening new academic venue for AI significance in consumer' insights.

## Data Availability Statement

The raw data supporting the conclusions of this article will be made available by the authors, without undue reservation.

## Author Contributions

HS provided the services for the proofreading as well as analysis of the article. MZ wrote the introduction and literature of the article. NZ wrote the methodology and discussion part of the article. All authors contributed to the article and approved the submitted version.

## Funding

This work was funded by Business School, Shandong Jianzhu University, China to HS.

## Conflict of Interest

The authors declare that the research was conducted in the absence of any commercial or financial relationships that could be construed as a potential conflict of interest.

## Publisher's Note

All claims expressed in this article are solely those of the authors and do not necessarily represent those of their affiliated organizations, or those of the publisher, the editors and the reviewers. Any product that may be evaluated in this article, or claim that may be made by its manufacturer, is not guaranteed or endorsed by the publisher.
